# Plasmonic Structure Enhanced Exciton Generation at the Interface between the Perovskite Absorber and Copper Nanoparticles

**DOI:** 10.1155/2014/128414

**Published:** 2014-09-11

**Authors:** Sheng Hsiung Chang, Kuen-Feng Lin, Chien-Hung Chiang, Sheng-Hui Chen, Chun-Guey Wu

**Affiliations:** ^1^Research Center for New Generation Photovoltaics, National Central University, No. 300 Jhongda Road, Jhongli, Taoyuan County 32001, Taiwan; ^2^Department of Optics and Photonics, National Central University, No. 300 Jhongda Road, Jhongli, Taoyuan County 32001, Taiwan

## Abstract

The refractive index and extinction coefficient of a triiodide perovskite absorber (TPA) were obtained by fitting the transmittance spectra of TPA/PEDOT:PSS/ITO/glass using the transfer matrix method. Cu nanoplasmonic structures were designed to enhance the exciton generation in the TPA and to simultaneously reduce the film thickness of the TPA. Excitons were effectively generated at the interface between TPA and Cu nanoparticles, as observed through the 3D finite-difference time-domain method. The exciton distribution is advantageous for the exciton dissociation and carrier transport.

## 1. Introduction

In recent years, mixed halide (CH_3_NH_3_PbI_3−*x*_Cl_*x*_) and triiodide (CH_3_NH_3_PbI_3_) perovskite absorber (PA) based photovoltaics have been intensively investigated because a power conversion efficiency (PCE) of 15% can be achieved by solution processes under low temperatures [1, 2]. There are several factors that can explain the high PCE. The bandgap of PA is about 1.64 eV [3], which can absorb half of the sun light. The exciton diffusion length of PA is longer than 1 micrometer [4]; therefore the bilayered structure based photovoltaics are workable [1–4]. The exciton binding energy of PA is about 50 meV [5], which results in good exciton dissociation at the interface between the PA and PCBM (Spiro-OMeTAD) [4]. The sharp optical absorption edge of PA corresponds to the small Urbach energy (~15 meV), which results in a high fill factor [6]. The thickness of PA has to be ~400 nm in order to efficiently absorb the incident sun light. However, a thicker PA is disadvantageous for exciton dissociation and carrier transport, limiting the photovoltaic performances in terms of short-circuit current density and fill factor. A PCE as high as 20% can be expected by improving the fill factor [7]. The above-mentioned drawbacks can be improved by using nanoplasmonic structures [8, 9]. Two degenerate transverse plasmon modes are supported by two-dimensional ordered Cu nanoplasmonic structure embedded in P3HT:PCBM blended film, which has been designed to enhance the absorption of P3HT:PCBM based inverted photovoltaics by 22% in the visible range [9]. In this work, the Cu nanoplasmonic structures were used to enhance exciton generation in the triiodide perovskite absorber (TPA) while simultaneously reducing the film thickness of TPA. The transfer matrix method (TMM) was used to calculate the transmittance, reflectance, and absorptance. The 3D finite-difference time-domain (FDTD) method was used to observe the plasmon-mediated exciton generation.

## 2. Optical Constants of Triiodide Perovskite Absorber

**Figure 1:**
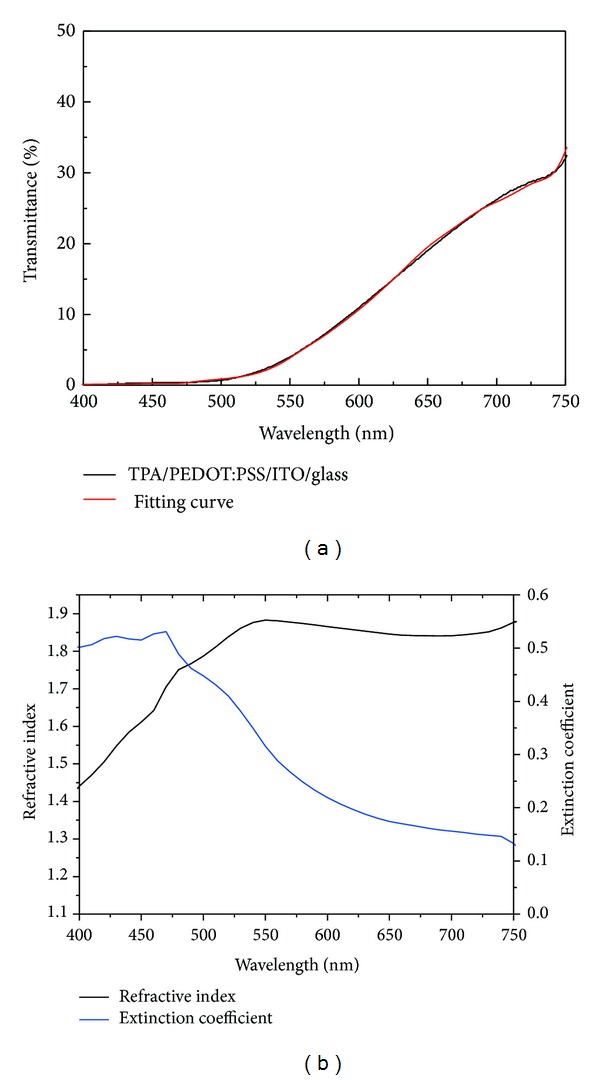
(a) Transmittance spectra of TPA/PEDOT:PSS/ITO/glass under normal incidence. (b) Refractive index and extinction coefficient of TPA film.

TPA was spin-coated on top of the PEDOT:PSS/ITO/glass by a sequential deposition method [1].  Figure 1(a) presents the transmittance spectrum of the TPA/PEDOT:PSS/ITO/glass. The transmittance spectrum was measured by a high accuracy spectrometer (Hitachi U-4100). The film thickness for each layer was measured by an *α*-step device (Veeco Dektak 150). The thicknesses of the TPA, PEDOT:PSS, and ITO were found to be 400 nm, 20 nm, and 250 nm, respectively. The refractive indices and extinction coefficients of the PEDOT:PSS thin film and the ITO film were taken from [9]. A Lorentz model was used to describe the dielectric constant of TPA, which can be written as follows:

(1) $$\begin{array}{c} \varepsilon_{\text{TPA}}\left(\omega \right)=\varepsilon_{b}+\overset{14}{\underset{n=1}{\sum }}\frac{s_{n}\omega_{L,n}}{\omega_{L,n}^{2}-\omega^{2}-jG_{n}\omega }, \end{array}$$

where *ε*_*b*_ (=1.5) is the background dielectric constant, *n* is the *n*th Lorentz pole, *s*_*n*_ is the strength, *ω*_*L*_ is the oscillation frequency, and *G* is the decay rate. Fourteen Lorentz oscillators were used in the fitting process. The transmittance spectrum of the TPA/PEDOT:PSS/ITO/glass was fitted using TMM. The fourteen oscillating wavelengths “*λ*_*L*_” were fixed and are listed in Table 1. *λ*_*L*_ = 2*πc*/*ω*_*L*_, where *c* is light speed in vacuum. The oscillation strengths and decay rates were scanned in the fitting process. An error function (EF) is defined to evaluate the accuracy between fitting and experimental curves. Consider

(2) $$\begin{array}{c} \text{EF}=\overset{750}{\underset{\lambda =400}{\sum }}\frac{\left|T_{\text{fit}}\left(\lambda \right)-T_{\exp}\left(\lambda \right)\right|}{351\times T_{\exp}}, \end{array}$$

where *T*_fit_ (red line) is the transmittance of the fitting curve and *T*_exp⁡_ (black line) is the measured transmittance of the TPA/PEDOT:PSS/ITO/glass. The value of the error function is equal to 0.025. The refractive index “*n*” and extinction coefficient “*k*” of the TPA film can be obtained by *n* + *ik* = (*ε*_TPA_)^1/2^. The optical constants of TPA are plotted in Figure 1(b). The fitted parameters are listed in Table 1.

## 3. Plasmonic Structure Enhanced Exciton Generation

**Figure 2:**
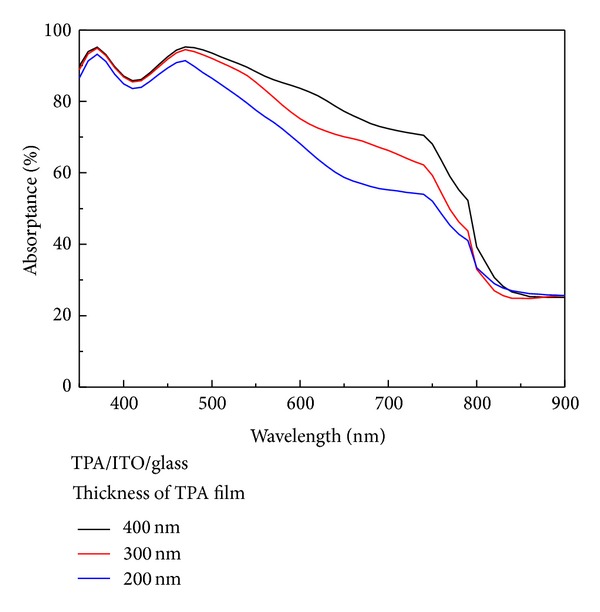
Absorptance spectra of TPA/ITO/glass for different TPA thicknesses.

**Figure 3:**
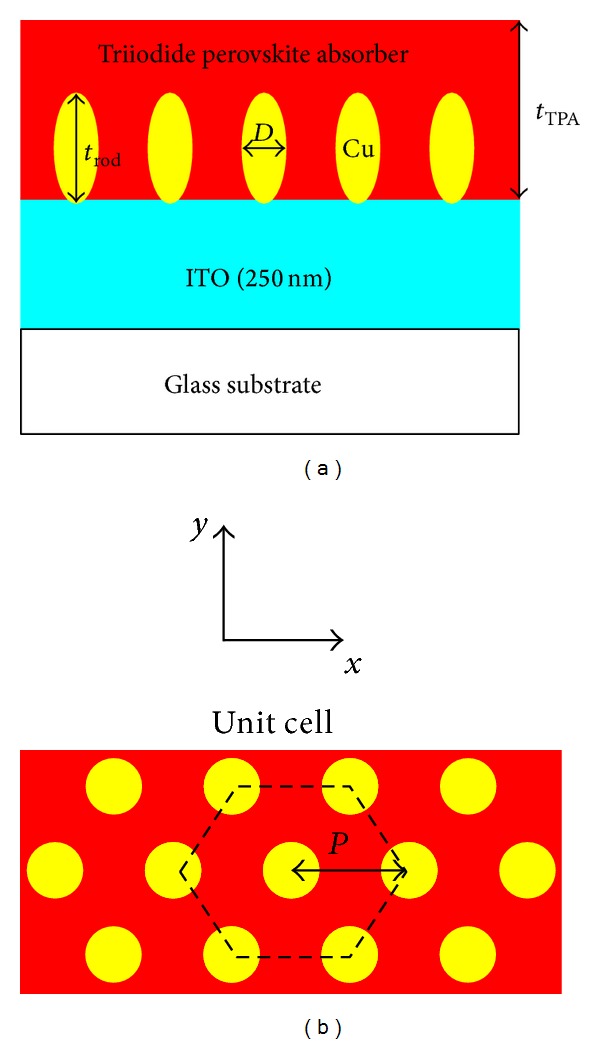
(a) Side view of TPA/ITO/glass with the Cu nanoplasmonic structure. (b) Top view of the Cu nanoplasmonic structure embedded in the TPA thin film.

Figure 2 presents the absorptance spectra of the TPA/ITO/glass, which was calculated using TMM. The absorptance of the TPA increases with an increase in thickness from 200 nm to 400 nm. In the wavelength range of 800 nm to 900 nm, the incident near-infrared light is absorbed by the ITO film due to the free carrier absorption. In order to reduce the thickness, a Cu nanoplasmonic structure was embedded in the TPA in order to enhance the absorptance in the effective absorption range (350 nm to 760 nm).  Figure 3 presents the Cu nanoplasmonic structure embedded in the TPA thin film. In this study, the period “*P*” was fixed at 100 nm. The gap size is defined by the difference between the period and the diameter of Cu nanoparticles.

The dipole-coupling model (DCM) [10] was adopted to calculate the effective dielectric constant of Cu nanoplasmonic structure embedded in the TPA film. These nanoplasmonic structures embedded can be treated as an effective medium. The physical concept of DCM is described in [9]. The Lorentz-Drude model was applied to Cu to calculate the refractive index and absorption coefficient [11].

**Figure 4:**
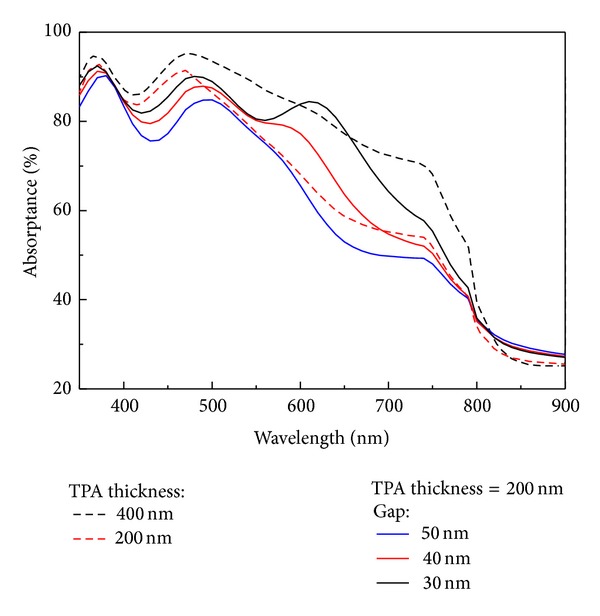
The red (black) dashed line indicates the absorptance of the TPA/ITO/glass when the TPA thickness is equal to 200 nm (400 nm). The blue, red, and black lines are the absorptance of the TPA/ITO/glass with the Cu nanoplasmonic structure when the long axis of the Cu nanoparticles is 100 nm.

**Figure 5:**
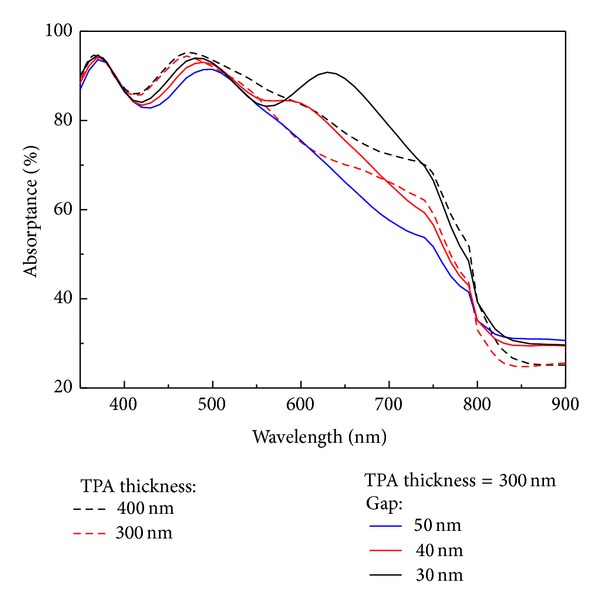
The red (black) dashed line indicated the absorptance of the TPA/ITO/glass when the TPA thickness is equal to 300 nm (400 nm). The blue, red, and black lines show the absorptance of the TPA/ITO/glass with the Cu nanoplasmonic structure when the long axis of the Cu nanoparticles is 150 nm.

Figure 4 presents the absorptance spectra of the TPA with and without the Cu nanoplasmonic structures. There was an increase in the absorptance of the TPA with Cu nanoplasmonic structures when the gap was changed from 50 nm to 30 nm. Compared with the red dashed line (TPA thickness = 200 nm) in Figure 4, the absorptance indicated by the black line is larger because the transverse plasmonic (TP) mode enhances the absorption (exciton generation) of the TPA. Compared with the black dashed line (TPA thickness = 400 nm) in Figure 4, the absorptance indicated by the black line is smaller. The absorptance spectra of a thicker TPA film with and without Cu nanoplasmonic structures are presented in Figure 5. Compared with the black dashed line (TPA thickness = 400 nm) in Figure 5, the absorptance indicated by the black line is higher due to the TP mode enhanced absorption even though the TPA thickness (=300 nm) is thinner. The Cu nanoplasmonic structure enhanced the absorptance of TPA in the effective absorption range (350 nm–760 nm) by 1.7% while reducing the TPA thickness from 400 nm to 300 nm.

## 4. Exciton Distribution

The 3D FDTD method was used to calculate the electric and magnetic field distributions of the Cu nanoplasmonic structures embedded in the TPA film. 20-cell perfectly matching layers were imposed at upper and lower boundaries to absorb the outgoing electromagnetic waves without producing significant reflections back into the simulation domain. The simulation of two-dimensional ordered Cu nanoparticle arrays was performed using periodic boundary conditions. The cell size and the time step used in discretization of the space domain and time domain were 1 nm × 1 nm × 1 nm and 1.9 × 10^−18^ s, respectively. A planewave with an *x*-directed electric field was launched from the glass substrate along the positive *z*-direction.

**Figure 6:**
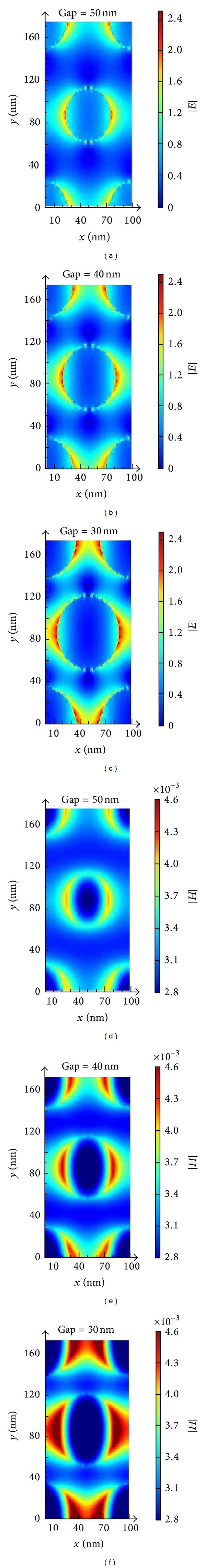
(a), (b), and (c) ((d), (e), and (f)) are the electric (magnetic) field distributions of the *x*-*y* plane for different gap sizes.

Figure 6 presents the field distributions of the *x*-*y* plane at the resonant wavelength of TP mode. The strengths of electric field and magnetic field both increased when the gap was changed from 50 nm to 30 nm. The incident light is trapped and redistributed in space to effectively generate excitons at the interface between the TPA and Cu nanoparticles. Conceptually, the excitons can be dissociated at the interface between TPA and Cu nanoparticles because the HOMO level (−5.4 eV) [3] of the TPA is lower than the Fermi level of Cu (−4.94 eV) [12]. Therefore, the localized field distribution benefits the exciton dissociation.

**Figure 7:**
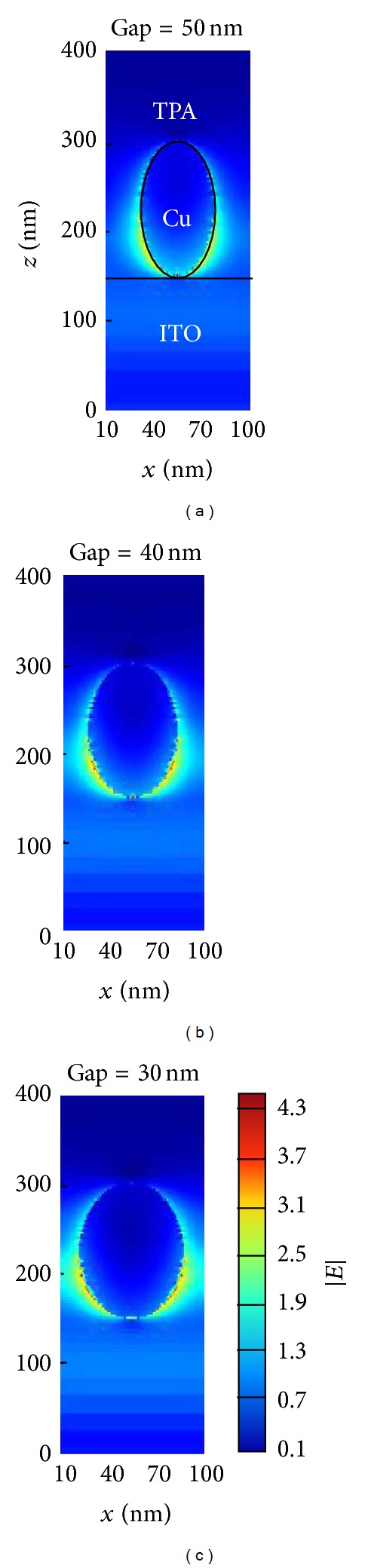
(a), (b), and (c) are the electric field distributions of the *x*-*z* plane for different gap sizes.

Figure 7 presents the electric field distributions of Cu nanoplasmonic structures embedded in the TPA film on the *x*-*z* plane. The electric fields (excitons) are localized (generated) around the lower surface of Cu nanoparticles. Therefore, the excitons are dissociated mostly from the lower surface of the Cu nanoparticles. After exciton dissociation, the holes (electrons) can propagate along the Cu (TPA) to the anode (cathode) electrode. In such cases, carrier recombination can be reduced.

## 5. Conclusions

In conclusion, we have assessed the optical effects of Cu nanoplasmonic structure embedded in triiodide perovskite absorber (TPA). The refractive index and absorption coefficient of TPA were obtained by fitting the transmittance spectrum of TPA/PEDOT:PSS/ITO/glass using transfer matrix method. Cu nanoplasmonic structures could reduce the TPA thickness from 400 nm to 300 nm while keeping the absorption strength. The 3D finite-difference time-domain method was used to observe the distribution of the electric field (generated excitons). The electric field is redistributed at the interface between the TPA and Cu nanoparticles, which benefits the exciton dissociation and carrier transport.

## Figures and Tables

**Table 1:** Fitted parameters of the Lorentz model for TPA film.

Oscillation wavelength (nm)	Strength	Decay rate (×10^14^/s)
*λ*_*L*,1_ = 380	*s*_1_ = 0.2	*G*_1_ = 10
*λ*_*L*,2_ = 430	*s*_2_ = 0.15	*G*_2_ = 8
*λ*_*L*,3_ = 470	*s*_3_ = 0.1	*G*_3_ = 6
*λ*_*L*,4_ = 500	*s*_4_ = 0.04	*G*_4_ = 4
*λ*_*L*,5_ = 520	*s*_5_ = 0.035	*G*_5_ = 3.5
*λ*_*L*,6_ = 540	*s*_6_ = 0.032	*G*_6_ = 3.5
*λ*_*L*,7_ = 570	*s*_7_ = 0.03	*G*_7_ = 4
*λ*_*L*,8_ = 600	*s*_8_ = 0.02	*G*_8_ = 4
*λ*_*L*,9_ = 630	*s*_9_ = 0.02	*G*_9_ = 4
*λ*_*L*,10_ = 670	*s*_10_ = 0.02	*G*_10_ = 3.5
*λ*_*L*,11_ = 710	*s*_11_ = 0.015	*G*_11_ = 3
*λ*_*L*,12_ = 720	*s*_12_ = 0.008	*G*_12_ = 3
*λ*_*L*,13_ = 730	*s*_13_ = 0.009	*G*_13_ = 2
*λ*_*L*,14_ = 745	*s*_14_ = 0.01	*G*_14_ = 1.5
